# Detection of Alpha-Methylacyl-CoA Racemase (AMACR), a Biomarker of Prostate Cancer, in Patient Blood Samples Using a Nanoparticle Electrochemical Biosensor

**DOI:** 10.3390/bios2040377

**Published:** 2012-09-26

**Authors:** Po-Yuan Lin, Kai-Lun Cheng, James D. McGuffin-Cawley, Fuh-Sheng Shieu, Anna C. Samia, Sanjay Gupta, Matthew Cooney, Cheryl L. Thompson, Chung Chiun Liu

**Affiliations:** 1 Department of Materials Science & Engineering, Case Western Reserve University, 10900 Euclid Avenue, Cleveland, OH 44106, USA; E-Mails: ppl3@case.edu (P.-Y.L.); jxc41@case.edu (J.D.M.-C.); 2 Department of Materials Science & Engineering, National Chung Hsing University, No. 250, Guoguang Road, Nan District, Taichung City 402, Taiwan; E-Mails: cklgraph1233@hotmail.com (K.-L.C.); fsshieu@dragon.nchu.edu.tw (F.-S.S.); 3 Department of Chemistry, Case Western Reserve University, 10900 Euclid Avenue, Cleveland, OH 44106, USA; E-Mail: axs232@case.edu; 4 Department of Urology, University Hospital Case Medical Center, Case Western Reserve University, 10900 Euclid Avenue, Cleveland, OH 44106, USA; E-Mail: sanjay.gupta@case.edu; 5 Division of Hematology/Oncology, University Hospital Case Medical Center, 10900 Euclid Avenue, Cleveland, OH 44106, USA; E-Mail: matthew.cooney@Uhhospitals.org; 6 Department of Family Medicine, University Hospital Case Medical Center, Case Western Reserve University, 10900 Euclid Avenue, Cleveland, OH 44106, USA; E-Mail: cheryl.l.thompson@case.edu; 7 Department of Chemical Engineering, Case Western Reserve University, 10900 Euclid Avenue, Cleveland, OH 44106, USA

**Keywords:** alpha-methylacyl-CoA racemase (AMACR), prostate cancer, biosensor, electrochemical, nanoparticles

## Abstract

Although still commonly used in clinical practice to screen and diagnose prostate cancer, there are numerous weaknesses of prostate-specific antigen (PSA) testing, including lack of specificity and the inability to distinguish between aggressive and indolent cancers. A promising prostate cancer biomarker, alpha-methylacyl-CoA racemase (AMACR), has been previously demonstrated to distinguish cancer from healthy and benign prostate cells with high sensitivity and specificity. However, no accurate clinically useful assay has been developed. This study reports the development of a single use, disposable biosensor for AMACR detection. Human blood samples were used to verify its validity, reproducibility and reliability. Plasma samples from 9 healthy males, 10 patients with high grade prostatic intraepithelial neoplasia (HGPIN), and 5 prostate cancer patients were measured for AMACR levels. The average AMACR levels in the prostate cancer patients was 10 fold higher (mean(SD) = 0.077 (0.10)) than either the controls (mean(SD) = 0.005 (0.001)) or HGPIN patients (mean(SD) = 0.004 (0.0005)). At a cutoff of between 0.08 and 0.9, we are able to achieve 100% accuracy in separating prostate cancer patients from controls. Our results provide strong evidence demonstrating that this biosensor can perform as a reliable assay for prostate cancer detection and diagnosis.

## 1. Introduction

Prostate cancer is the most common malignancy among men in the United States and also ranks as the second most common cause of cancer death in males [1]. It is estimated to be diagnosed in over 200,000 men in 2012, with over 28,000 deaths attributed to this cancer [2]. The prostate specific antigen (PSA) blood test in addition to the digital rectal exam, have traditionally been the preferred modalities to screen for prostate cancer [3,4,5,6,7,8,9,10]. While prostate cancer screening leads to an early diagnosis of prostate cancer, which in turn permits curative treatment, it also has significant limitations because serum PSA is not specific to prostate cancer [7]. One of the major limitations of PSA screening is that serum PSA can be elevated in patients with other common benign conditions, such as benign prostatic hyperplasia, prostatitis, or after minor clinical procedures such as transrectal ultrasound [11,12,13,14,15]. Therefore, approximately for every four men that have prostate biopsies, one case of prostate cancer is detected [16,17,18]. Currently the United States Preventative Service Task Force (USPSTF) does not recommend prostate cancer screening using PSA. The USPSTF position on prostate screening is the result of the inability of using PSA to demonstrate a reduction in the risk of death.

PSA is also not a reliable biomarker for aggressive prostate cancer. High grade prostate cancers may actually produce less PSA and the absolute PSA number does not accurately reflect the aggressiveness of disease [19,20,21,22]. Many of the men diagnosed with prostate cancers have clinically insignificant disease, which will never become symptomatic in their lifetime. This “over-diagnosis” of clinically insignificant prostate cancer from PSA screening has been estimated to be as high as 30% with subsequent over-treatment [23,24,25]. The side effects of prostate cancer treatment may include unnecessary painful biopsies, surgical complications, radiation burns, incontinence, erectile dysfunction, bowel injury, and patient anxiety [3,23,25]. Currently, there is no recommended screening method for prostate cancer. Thus, there is a clear need for an improved biomarker for prostate cancer able to distinguish between indolent and aggressive disease [9,10].

Alpha-methylacyl-CoA racemase (AMACR) is an enzyme involved in peroxisomal beta-oxidation of dietary branched-chained fatty acids. AMACR has been consistently overexpressed in prostate cancer epithelium; hence it becomes an ideal specific biomarker for cancer cells within the prostate gland [3,5]. Over-expression of AMACR may increase the risk of prostate cancer, because its expression is increased in premalignant lesions (prostatic intraepithelial neoplasia) [3,5]. Furthermore, epidemiologic, genetic and laboratory studies have pointed to the importance of AMACR in prostate cancer [26,27]. Genome-wide scans of linkage in hereditary prostate cancer families have demonstrated that the chromosomal region for AMACR (5p13) is the location of a prostate cancer susceptibility gene [9,10] and AMACR gene sequence variants (polymorphisms) have been shown to co-segregate with cancer of the prostate in families with hereditary prostate cancer [7].

AMACR is detectable in the urine of men who have a prostatic biopsy [6]. In a previous study sera from men with biopsy-proven prostate cancer and men without known prostate cancer have been screened for a humoral immune response to AMACR [8]. This study concludes that AMACR immune-reactivity is statistically significantly higher in the sera from cancer case subjects than from the control subjects. These studies suggest that if a reliable method for the detection of AMACR from blood or urine can be established, it will be able to identify men with prostate cancer and may be able to serve as a reliable biomarker for prostate cancer.

In this study, we present the results of the development of a biosensor for the detection of AMACR in human serum samples. We test our biosensor for the ability to identify patients with prostate cancer. We find that, using this biosensor with plasma samples from 24 men, we were able to distinguish, with 100% accuracy, between both healthy men, men with high grade prostatic intraepithelial neoplasia, and men with biopsy proven prostate cancer. 

## 2. Experimental

### 2.1. Chemistry and Reaction Mechanisms of Detecting AMACR

A reaction mechanism involving AMACR has been suggested by Lloyd [28]. We recognize from this reaction mechanism that if pristanic acid is used as a reaction substrate, the following reaction mechanisms occur.

**Figure 1:**
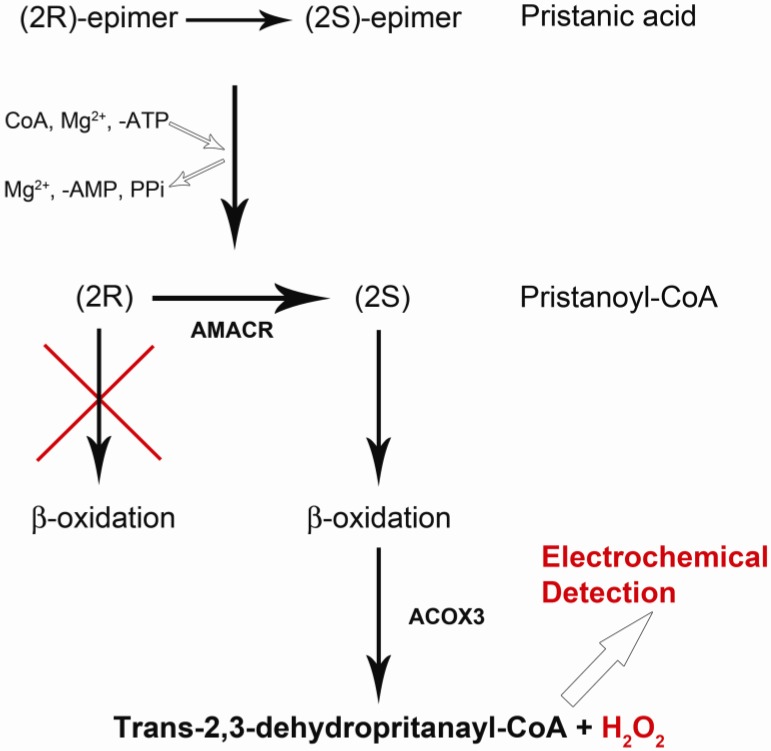
Pathway from Pristanic Acid to Pristanoyl-CoA then to producing H_2_O_2_.

Pristanic acid possess four methyl groups [28,29]. Based on the reaction mechanism in Figure 1, pristanic acid can be employed as reaction substrate, which consists of two epimers designated as (2*R*) and (2*S*). Both (*2R*) and (*2S*) epimers can react with proper quantities of coenzyme A (CoA), ATP and Mg^2+^ in the presence of very long chain fatty acid-coenzyme A (VLCFA-CoA) synthetase forming (*2R*)-pristanoyl-CoA and (*2S*)-pristanoyl-CoA, respectively. However, the (*2R*)-pristanoyl-CoA cannot carry out the b-oxidation process. On the other hand, (*2S*)-pristanoyl-CoA in the presence of the enzyme ACOX3 (peroxisomalacyl-coenzyme A oxidase 3) can carry out the b-oxidation process producing H_2_O_2_. H_2_O_2_ can be oxidized electrochemically generating a current which can then be used to quantify (*2S*)-pristanoyl-CoA. AMACR converts (*2R*)-pristanoyl-CoA to (*2S*)-pristanoyl-CoA, resulting in a higher H_2_O_2_ level, and the oxidation current of H_2_O_2 _can then be used to quantify the amount of AMACR present. 

### 2.2. AMACR Biosensor Fabrication

**Figure 2:**
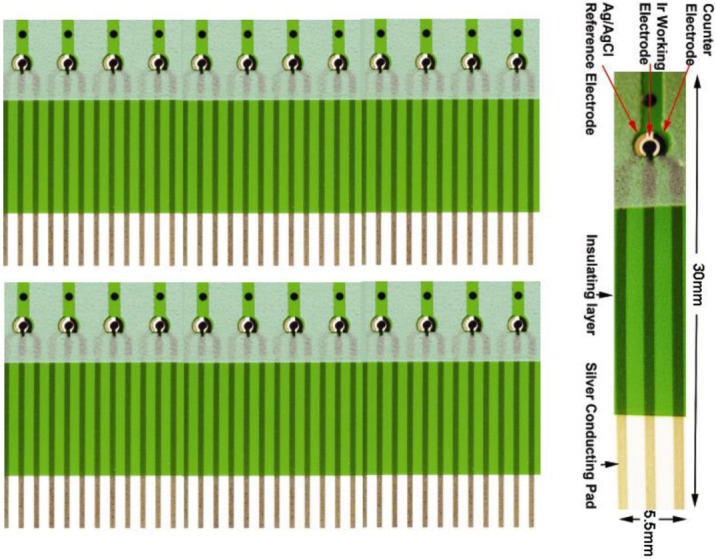
Screen-printed single-use, disposable Ir nano-catalyst contained H_2_O_2_ platform biosensor prototype.

This biosensor was thick film printed on 0.18 mm thick polyethylene terephalate (PET) substrate (Melinex 329, DuPont Co.) in the dimension of 385 × 280 mm^2^. The cost of this thick film process was relatively low, and 150 individual biosensors were produced in 6 rows per sheet. The biosensor had a three-electrode configuration: working, counter and reference electrode. Both the working and counter electrodes were printed with nano-particle of iridium (actually IrO) contained active carbon power (RC72), which was mixed into a thick-film printing ink. The reference electrode was also a thick film printed Ag/AgCl electrode using DuPont #5870 Ag/AgCl thick film ink. The insulation layer was thick-film printed using Nazdar APL 34 silicone-free dielectric ink (colored in green in Figure 2), which also defined the dimensions of the individual biosensor. The working electrode was approximately 0.8 mm^2^ with a diameter of 1.0 mm. The biosensor could accommodate 10–20 micro-liter of test volume. Other electrocatalysts have been tested and evaluated in addition to IrO nano-catalyst. However, the test results are not suitable to be discussed here. We will report only the use of IrO nano-catalyst in this study which provides most promising results. It must be recognized that because this biosensor prototype was fabricated by thick-film printing technology, the manufacturing cost can be relatively low thereby making a single use, disposable biosensor a reality. However, thick film printing technology has an inherent accuracy limitation of 10%. Therefore, this limitation must be included in the consideration of the biosensor measurement. 

Figure 2 shows the biosensor prototype, which was developed in our laboratory. Using this unique H_2_O_2_ biosensor and the AMACR assay approach described in Figure 1, we are able to establish an *in vitro* assay of AMACR in serum samples for effective prostate cancer detection and diagnosis.

### 2.3. Calibration of this AMACR Biosensor

**Figure 3:**
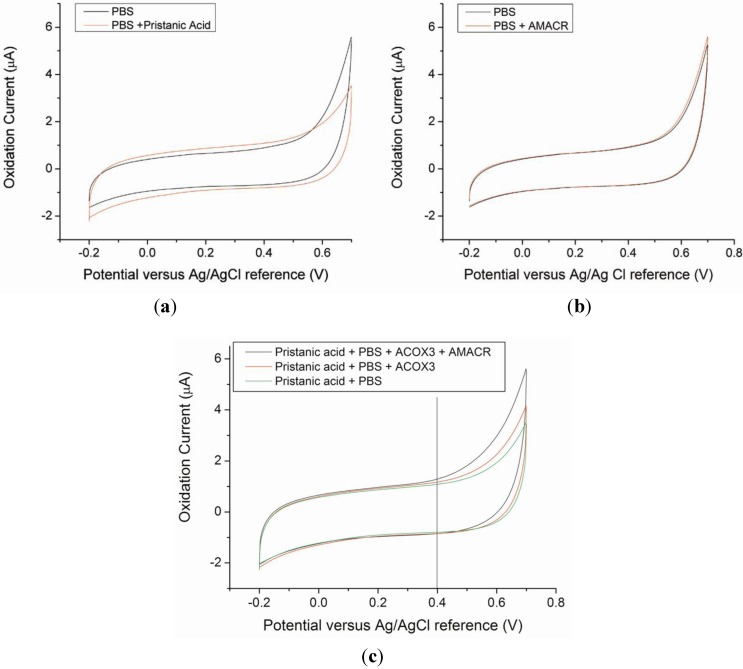
Cyclic Voltammograms of control sample solutions of PBS media, PBS + pristanic acid (**a**), and PBS + AMACR (0.0065 µg/µL) (**b**), The CVs obtained show that pristanic acid and AMACR do not contribute to any current measured by the Ir-nanoparticle based prototype biosensor. (**c**) Cyclic Voltammograms obtained from test solutions containing pristanic acid, in the absence and presence of the enzymes ACOX3 and AMACR, respectively.

Hydrogen peroxide, H_2_O_2_, is an electrochemically active species, which can be oxidized or reduced under appropriate conditions. Normally, at approximately +0.40 to 0.45 V *versus* a Ag/AgCl reference electrode, H_2_O_2_ can be oxidized yielding an oxidation current which corresponds to the quantity of H_2_O_2_ present. With the incorporation of the IrO nano-catalyst, the overpotential of the oxidation of H_2_O_2_ becomes lower and H_2_O_2 _in phosphate buffer solution (PBS). This represents the advantage and uniqueness of this biosensor. The sensitivity of the IrO catalyst biosensor for H_2_O_2_ detection in PBS has been reported elsewhere [30], and the biosensor is used in the AMACR measurement as shown in Figure 1. However, there is no direct calibration of H_2_O_2_ using AMACR contained samples, because the H_2_O_2_ generated in the patient blood samples is low comparing the calibration of H_2_O_2_ in PBS. The calibration of the biosensor is carried out directly using the blood samples of the patients and the testing solutions of known AMACR. The detection approach and the reaction mechanism shown in Figure 1 can only be applicable for AMACR detection, if all the chemical species involved in the reactions will not contribute to any oxidation current of the H_2_O_2 _produced. This can be validated by carrying out the cyclic voltammetric studies of the chemicals involved. Figure 3 shows the cyclic voltammetric studies indicating that the chemicals used will not contribute to the oxidation current of hydrogen peroxide produced in Figure 1. 

Figure 3(a) shows the background electrochemical signal from the AMACR substrate, pristanic acid, in phosphate buffer saline (PBS), demonstrating that the substrate by itself does not contribute to any background current. Similarly, the enzyme, AMACR, does not contribute to the background current. Figure 3(b) shows the absence of detectable background current due to AMACR (0.0065 µg/µL) in PBS media. From the metabolic b-oxidation pathway of pristanic acid illustrated in Figure 1, (*2S*)-pristanoyl-CoA cannot be oxidized without the presence of ACOX3 (*i.e.*, electrochemically detectable H_2_O_2_ will not form without ACOX3). This is verified in Figure 3(c), where the experimental results of the oxidation currents detected by the biosensor in the presence of pristanic acid, pristanic acid + ACOX3, and pristanic acid + ACOX3 + AMACR are shown. This increase in detected oxidation current due to the addition of AMACR is specific and unique for pristanoyl-CoA and free of interferences from other molecules. In Figure 3(c), the higher current produced as a result of the conversion of (*2R*)-pristanoyl-CoA by AMACR to (*2S*)-pristanoyl-CoA is evident. These results illustrate that the electrochemical detection of AMACR is quantitative and selective.

This reaction required an incubation time period to produce H_2_O_2_. Various incubation times were used and assessed. We have chosen 48 h as the incubation time, and this length of time may be further optimized.

## 3. Results and Discussion

To test the practical application of this AMACR biosensor, measurement of the level of AMACR in human biological specimens was carried out. We expected that the level of AMACR would increase in prostate cancer patients. In order to evaluate this assessment, plasma samples from 9 healthy males, 10 men with high grade prostatic neoplasia (HGPIN) and 5 men with prostate cancers were used in a laboratory-blinded test of the AMACR levels in these samples. 5mL of blood was collected from each patient in standard heparinized tubes. Plasma was isolated by standard protocols and frozen until future use. All samples were collected prior to treatment, where relevant. 

Table 1 shows the characteristics of these patients and the average AMACR levels. To quantify AMACR, samples were first thawed and 5 µL aliquots were made. Pristanic acid (#P6617 Sigma-Aldrich) was mixed with phosphate buffer solution (PBS) with a volumetric ratio of 1:1. PBS solution had a pH value of 6.5 prepared by mixing the appropriate quantity of monobasic and dibasic sodium phosphates with deionized water. 200 mM of potassium chloride was added into the PBS as the supporting electrolyte improving the conductivity of the buffer solution. 3 mg of adenosine triphosphate ATP (#A1852 Sigma-Aldrich) 3 mg magnesium chloride (#208337 Sigma-Aldrich) and 3 mg coenzyme A (CoA) (#C3144 Sigma-Aldrich) were added into the a total of 140 µL of the pristanic acid-PBS mixed solution. This solution was incubated in −20 °C for 72 h prior to use in any testing. 

**Table 1:** Population description and mean AMACR levels by patient group.

	Healthy Controls (N = 9)	HGPIN (N = 10)	Prostate Cancer Cases (N = 5)
Gleason score, N (%)	N/A	N/A	
3 + 3			4 (80%)
3 + 4			1 (20%)
Mean (SD) Plasma PSA, ng/mL	2.31 (1.67)	18.86 (7.43)	15.81 (11.43)
Mean (SD) Plasma AMACR, µg/µL	0.005 (0.001)	0.0004 (0.0005)	0.077 (0.10)

The applied potential for the current measurement was set at +0.4 V *versus* the Ag/AgCl reference. 5 µL of the prepared pristanic acid-PBS solution was first mixed with 1 µL of peroxisomalacyl-coenzyme A oxidase 3 (ACOX3) (#H00008310-Q01 Sigma-Aldrich) and then mixed with 1 µL of the human serum sample. This mixture was incubated for one hour at room temperature (~21 °C). 5 µL of this mixture was then drawn and placed on the biosensor surface and the oxidation current measured. All samples were run in triplicate. Laboratory personnel were blinded to the disease status of the samples. 

The mean of the triplicate runs for each sample was calculated to represent the estimated quantity of AMACR in that sample. Sensitivity and specificity were calculated using a cutoff as determined by the data.

**Figure 4:**
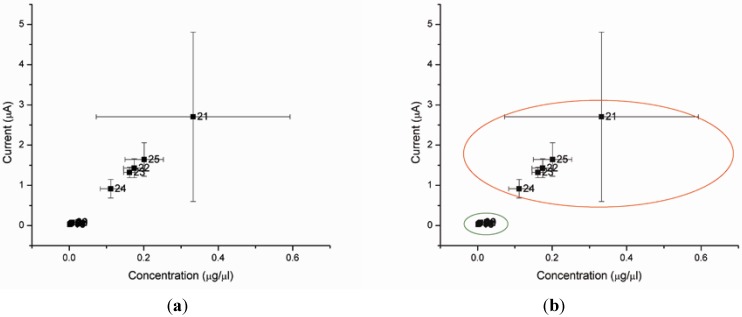
(**a**,**b**) Biosensor reading from the plasma of 24 test subjects. Samples 1–9 are from healthy men. Samples 11–20 are from men with HGPIN. Samples 21–25 are from men with prostate cancer (Gleason score 6–7). (Note: Sample 10 is not available).

Figure 4 shows the level of AMACR as measured by our biosensor for each sample. As illustrated in Figure 4(a), using a current level cutoff of anywhere between 0.08 and 0.90 would provide 100% sensitivity and 100% specificity to separate the prostate cancer cases from the other patients. Thus, in our preliminary data, the accuracy of this test is 100%. 

## 4. Conclusions

In this study, we apply our nano-particle metallic catalyst electrochemical biosensor to detect a previously reported prostate cancer biomarker, AMACR. We have demonstrated that our biosensor has 100% accuracy. Previous studies have detected AMACR in 137 prostate cancer patients and reported 100% accuracy [31]. Importantly, unlike the PSA, this detection method clearly distinguishes prostate cancer patients from not only from healthy males but also from men with high grade prostatic intraepithelial neoplasia (HGPIN). 

We recognize that the sample size tested is relatively limited at this stage. Additional prospective studies will need to be undertaken to further evaluate the potential of this sensor to aid in prostate cancer screening and diagnosis. 

This detection technique could only be applied effectively if the reaction mechanism, the potential interference species, the biosensor design and detecting electrode material were well defined and experimentally verified, as we have demonstrated here. Of practical clinical importance, this biosensor is designed and fabricated as a single use, disposable device, though it requires approximately forty hours of incubation time for the operation of the biosensor. The length of this incubation time required can be optimized in further study. This biosensor is relatively cost-effective and inexpensive. In a practical application, this biosensor will be incorporated into a hand-held potentiostat current output reading meter, similar to the conventional glucose meter, as a simple and minimally invasive clinical assay. 

In summary, we have developed a biosensor that can very accurately detect AMACR and thus detect the presence of prostate cancer. Future studies are needed to evaluate this biosensor on large scale population for the screening and to understand how this detection method relates to different clinical stages of prostate cancer. Our sensor only requires a very small amount (approximately a drop, less than 2 µL) of bodily fluid. An even less invasive use of this sensor would be using a needle prick of whole blood and/or a drop of urine. However, the application of this biosensor and the detection technique using other human fluids, such as urine or whole blood, will need to be further examined. Future projects will lead to wider assessment of this technology.

Importantly, this detection method clearly distinguishes prostate cancer patients not only from healthy men, but also from men with HGPIN, which is a common limitation of other prostate cancer biomarkers. The current output of this detection method is one order of magnitude higher for prostate cancer patients compared to that for either healthy or HGPIN males. This large difference in current outputs for the biosensors provides accuracy in distinguishing the prostate cancer patients from the normal and benign individuals. Although it will be necessary to quantify AMACR levels in a larger number of patients with different stages of cancers and correlate potential similar background factors for prostate cancers before a definitive assessment can be made, the findings thus far demonstrate the detection of AMACR level using this relatively simple and minimally-invasive (not requiring a biopsy) method is very accurate.
